# Perceptions of and barriers to ethical promotion of pharmaceuticals in Pakistan: perspectives of medical representatives and doctors

**DOI:** 10.1186/s12910-020-00569-0

**Published:** 2021-01-04

**Authors:** Rehan Gul, Hamid Saeed, Zikria Saleem, Fawad Rasool, Furqan Kurshid Hashmi, Muhammad Islam, Imran Imran, Syed Atif Raza, Zeeshan Danish

**Affiliations:** 1 College of Pharmacy, Universality of the Punjab, Allama Iqbal Campus, Lahore, 54000 Pakistan; 2 Department of Pharmacy, University of Lahore, Lahore, Pakistan; 3 Department of Pharmacy Practice, Faculty of Pharmacy, Bahauddin Zakariya University, Multan, Pakistan; 4 Department of Pharmacology, Faculty of Pharmacy, Bahauddin Zakariya University, Multan, Pakistan

**Keywords:** Pakistan, Sales, Marketing, Ethical, Promotion, Un-ethical, Pharmaceuticals, Medical representative, Medical doctor

## Abstract

**Background:**

In Pakistan, drug promotion practices, ethical or unethical, have rarely been in the spotlight. We aimed to assess the perception and barriers of medical representatives (MRs) and doctors (MDs) regarding ethical promotion of pharmaceuticals in Pakistan.

**Methods:**

A cross sectional survey was conducted in seven major cities of Pakistan for 6-months period. Self-administered questionnaire was used for data collection. Logistic regression and five-point Likert scale scoring was used to estimate the perceptions and barriers.

**Results:**

Compared to national companies (NCs), the medical representatives (MRs) of multinational companies (MNCs) strongly believed that their companies follow World Health Organization (WHO) (OR; 5.31, *p* = 0.0005), International Federation of Pharmaceutical Manufacturers & Associations (IFPMA) (OR; 6.45, *p* = 0.0005) and national codes of ethics (OR; 5.84, *p* = 0.0005). MNCs trained their MRs (OR; 6.68, *p* = 0.0005), provide accurate and valid scientific data (OR; 4.01, *p* = 0.007) with adequate system of accountability and controls on product samples (OR; 1.96, *p* = 0.047), while, NCs sponsor social or entertainment activities, seminars and conferences, and all sort of facilitation in form of gifts of their choice and clinic renovation for medical doctors (MDs). MDs perceptions were similar to MRs mentioned above, yet strongly agreed that companies offer cash payments or equivalents to MDs. The MRs of NCs/MNCs and MDs agreed/strongly agreed that no external accountability, profiteering, pressure on sale targets, job insecurity, condoning unethical promotion by high-ups’ and business promotion by junior MDs were the predominant barriers.

**Conclusion:**

In conclusion, MRs of MNCs and MDs believed that MNCs follow certain codes of ethics in the promotion of pharmaceuticals, while NCs tend to be more profit oriented and even condone unethical promotion. All stakeholders, MRs, MDs and companies, might pose certain barriers, intentionally or unintentionally, in ethical promotion.

## Background

In different parts of the world, the interpretation of the term “ethical” varies in societies, which pertains to or deals with the morals or principles of morality, while the term “promotion” refers to all the informational and persuasive activities by the companies. In line with this, the ethical criteria for drug promotion should be based on the proper behaviors that are consistent with the search for truthfulness and righteousness. According to World Health Organization (WHO) 2018 estimates, the global pharma market is worth $1.4 trillion per annum [1] across the globe. In Pakistan, as of today, approximately 620 pharmaceutical companies are registered with Drug Regulatory Authority of Pakistan (DRAP), out of which less than 30 are multinational companies (MNCs) and the rest are national companies (NCs), where 2/3rd market share is clutched by MNCs while NCs enjoy the remaining 1/3rd [2]. According to International Federation of Pharmaceutical Manufacturers and Associations (IFPMA), Pakistan’s total pharmaceutical sales is estimated at $2.29 billion—among others, $1.70 billion of prescription drugs and $0.59 million of over the counter (OTC) drugs [3].

Pharmaceutical drug promotion, a term used to entail all the communicative and persuasive efforts by pharmaceutical manufacturers and distributors to invoke pharmaceuticals demand [4]. Drug promotion is pivotal in galvanizing drug sales and in doing so may impact the rational use of drugs, drug price controls, manufacturing, availability, equity of drug distribution and overall cost of health care system [5]. All over the world, including Pakistan, pharmaceutical companies promote their drugs to doctors, patients and health care facilities through medical representatives (MRs)—often science (medical or biology) graduates. In 2015, the California based Institute for Health and Socio-economic Policy reported that out of top 100 pharmaceutical companies by sales, 64 spent twice the amount on marketing and sales than on Research and Development (R&D), 58 spent three times, 43 spent five times and 27 spent ten times the amount [6]. Therefore, to boost the sales and to achieve their assigned targets, MRs use diverse marketing gimmicks, such as use of drug samples, exclusive giveaways with embossed or printed names of target drugs in form of prescribing pads, pens and coffee mugs, in an attempt to inscribe and prioritize the names of target drug on prescriber’s inmost subconscious mind [7]. In Pakistan, this interaction even extends to financial assistance in form of refurbishing doctor’s offices, sponsored visits to international conferences (sometimes families included), sponsoring conferences organized by doctor’s associations and even sponsoring brand new leased cars [8, 9].

In Pakistan, the term “unethical promotion” is a well-known practicing fact in the realm of pharmaceutical marketing, chiefly created and practiced by multinational companies (MNCs) because of adequate finances to afford and support these practices [10]. Following MNCs, the national companies (NCs) resort to the same kind of practices, even more intensified, because the local products cannot compete with MNC’s product in quality, efficacy, and safety, though with few exceptions. A study from Pakistan stated that both the MDs as well as pharmaceutical companies and their representatives are responsible for unethical promotion of the pharmaceuticals [11]. Besides, pharmaceutical companies who hesitate to offer money dividends to the doctors often failed to get the prescriptions for their brand [12]. Thus, the previous reports and the current drug promotion practices indicate that the un-ethical drug promotion has become an acceptable norm of the Pakistan’s pharmaceutical industry, patronized and practiced with sense of complacency by major stakeholders, i-e., doctors, government, pharmacists, and health regulators at the expense of patient welfare. There is scanty of literature evidences about the perception of MRs and MDs regarding ethical promotion of pharmaceuticals and posed barriers in Pakistan. Thus, we aimed at conducting this very first study in Pakistan to estimate the perception of and barriers to ethical promotion of pharmaceuticals in Pakistan from two major stakeholders—the MRs and the MDs.

## Methods

### Study design

**Fig. 1:**
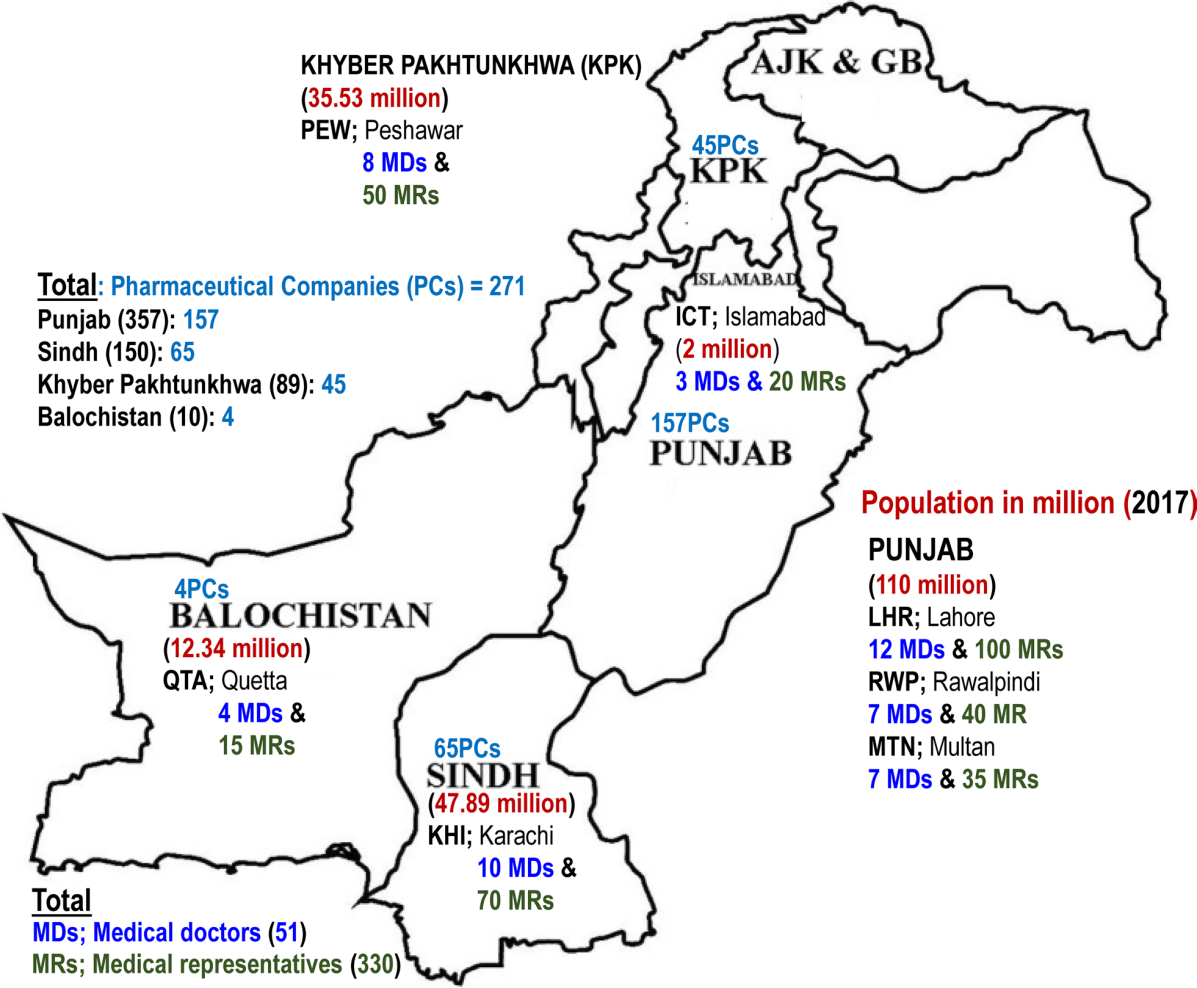
A brief overview of the number and location of sampled pharmaceutical companies (PCs) medical representatives (MRs) and medical doctors (MDs) from 7 major cities of all four provinces of Pakistan. Population of each province is mentioned in red as per 2017 census. Numbers indicated in map are the actual numbers considered for final analysis. The free printable blank map of Pakistan was taken from www.blankworldmaps.com and was adapted for the figure

A cross-sectional survey base study was conducted in 7 major cities of all four provinces, Punjab (Lahore, Rawalpindi and Multan), Khyber Pakhtunkhwa (Peshawar), Sindh (Karachi) and Baluchistan (Quetta) and federal capital city (Islamabad) of Pakistan from January 02, 2018 to July 30, 2018. Data was collected from both, medical representatives (MRs) of national companies (NCs) and multi-national companies (MNCs), along with medical doctors (MDs). List of 609 registered national and multi-national pharmaceutical companies were acquired from drug regulatory authority of Pakistan (DRAP, www.dra.gov.pk, 2018)—357 in Punjab, 150 in Sindh, 89 in Khyber Pakhtunkhwa and 10 in Baluchistan. Only 554 registered companies were considered as rest of the companies were either of veterinary medicines or cotton/bandages. Out of 554, only 271 pharmaceutical companies accepted to participate in the study. Thus, for data collection, questionnaires were sent to 271 pharmaceutical companies, NCs = 217 and MNCs = 54, at their drug distribution setups, since MRs and the managers use these offices for official meetings. The final enrollments consisted of 205 MRs from NCs and 125 from MNCs. The province and city wise enrollments of pharmaceutical companies are mentioned in Fig. 1.

For MD’s perspective, GP’s and specialist were enrolled from community clinics and teaching hospitals, respectively, of the 7 cities. MDs were identified via Pakistan Medical and Dental Council (PMDC), the statutory, regulatory and registration authority for medical and dental education, and practitioners of Pakistan, registered database upon an official request and were approached via letters of request to participate in the study.

### Study population

Sample size of MRs was calculated by estimating total number of pharmaceutical companies and extracting data on average number of products assigned to each MR. On an average 3–6 products were assigned to each MR to promote in one specific zone of a city. Thus, we estimated an average of 6 MRs for one city (making 6 zones; I MR for 1 zone) by one pharma company. This makes a workforce of almost 42 MRs, excluding managers, in 7 major cities of Pakistan by one pharma company. For 554 pharmaceutical companies, considering an average of 42 MRs per company, the estimated number of MRs working in 7 major cities of Pakistan were almost 23,268. Using Rao-soft sample size calculator (http://www.raosoft.com/samplesize.html), assuming a population of 23,268 with a confidence interval of 95% and margin of error of 5%, the study sample was found out to be 378. For an estimated response rate of 70% a total of 542 medical representatives were targeted for the distribution of questionnaires. The number of MRs and MDs, province and city wise, included in final analysis based on consent to participate and completely filled questionnaires as summarized in Fig. 1.

### Medical representatives (MRs)

A total of 542 questionnaires were distributed to pharmaceutical companies (*n* = 271), 2 questionnaires per company to enroll MRs under the following criteria;

*Inclusion criteria;* all MRs, irrespective of age, gender, ethnicity, with bachelor’s degree, minimum working experiences of 1 year, and willing to participate in the study were included in the study.

*Exclusion criteria;* MRs not having bachelor’s qualification, less than 1 year of working experience, returned half-filled questionnaires and not willing to participate in the study were excluded from the study.

Out of 542 distributed questionnaires, 128 questionnaires were partially filled, and 84 questionnaires were not returned back. Thus, a total of 330 MRs were included in the study for data analysis (Fig. 1)—a response rate of 61%.

### Medical doctors (MDs)

A systematic scheme based on population and number of public hospitals, was used to enroll MDs from 7 major cities. Based on population, for Karachi, Lahore, Multan, Peshawar and Rawalpindi, 3 major tertiary care public hospitals and 3 GP clinics were included from each city. Thus, 3 specialist doctors from three tertiary care hospitals (3 × 3 = 9) and 3 general practitioners were enrolled—12 MDs from each city to make a total of 60 MDs from 5 major cities. For Islamabad and Baluchistan, 2 tertiary care public hospitals and 1–2 GP clinics were included from each city, thus, 3 specialist doctors from each tertiary care hospital (2 × 3 = 6) and 1–2 GPs were enrolled—7 MDs from each city to make a total of 15 MDs from these 2 cities. Thus, in total 75 questionnaires were distributed among MDs as per study inclusion and inclusion criteria.

*Inclusion criteria;* the specified number of registered MDs of teaching hospital, medical specialist (FCPS part 1 or 2, Fellow of College of Physicians and Surgeons Pakistan (FCPS) is a postgraduate qualification awarded by the College of Physicians and Surgeons Pakistan upon completing specialized training in chosen area of specialization, almost 73; one has to complete part 1 before completing part 2), and GPs both having at least 10 years of practice and willing to participate in the study were included in the study.

*Exclusion criteria;* non-registered MDs, those not registered with PMDC, registered MDs with working experience of less than 10 years and not willing to participate in the study were excluded from the study.

All the returned questionnaires were completely scrutinized to exclude the partially filled questionnaires, thus, only 51 completely filled questionnaires were used for data analysis (Fig. 1)—a response rate of 68%.

### Data collection

The questionnaires, for MR and MD, were designed keeping the guidelines on ethical promotion of pharmaceutical products by IFPMA (Appendix 1). Questionnaires were validated by expert academician for content validation and suggestions were incorporated to make it more objective driven. Face validation was done by conducting a pilot study on 25 enrollees. Questionnaire for MRs and MDs were comprised of demographics, perception about ethical promotion and self-perceived posed barriers in ethical promotion of pharmaceuticals in Pakistan. Self-administered questionnaires were distributed among both, MRs and MDs with empty envelops with postal stamps to ensure their confidentiality and timely return to the field administrators.

### Data analysis

Data was analyzed by using statistical software SPSS. The percentages and frequencies were estimated using descriptive statistics. Inferential data was analyzed by using cross-tabulation and associations were determined using Pearson’s chi-square. Bivariate logistic regression was used to determine the odd ratios. Data was presented in the form of tables. An alpha value of 0.05 or less was considered statistically significant.

## Results

### Demographics of medical representatives and doctors

The demographics of MRs and MDs are summarized in Table 1. Majority of MRs were males (88.84%), working in NCs (NCs; 62.12%, MNCs; 37.88%) had Bachelor of Science degree (58.18%) and were hailing from Punjab (61.2%), while only 10% MRs had Pharm D education. More than 50% MRs claimed that they paid 10–15 visits per day to their customers (MDs) and more than 80% were satisfied with their jobs (Table 1).

**Table 1:** Demographics of medical representatives (MRs) and medical doctors (MDs)

Characteristic of medical representatives	Frequency (*n* = 330)	Percentage (%)
*Gender*		
Male	292	88.48
Female	38	11.52
*Company*		
National (NC)	205	62.12
Multi-national (MNC)	125	37.88
*Terminal education*		
Bachelors	192	58.18
Master	105	31.81
Pharm D	33	10.00
*Provincial region*		
Punjab	202	61.2
Sindh	57	17.3
Khyber Pakhtunkhwa	43	13
Baluchistan	8	2.4
Federal Area	20	6.1
*Number of visits to health care providers/day*		
< 5	19	5.76
5–10	93	28.18
11–15	181	54.85
> 15	37	11.21
*Satisfied with drug promotion activities*		
No	52	15.76
Yes	278	84.24

Characteristics of medical doctors	Frequency (*n* = 51)	Percentage (%)
*Gender*		
Male	45	88.24
Female	6	11.76
*Practicing facility*		
Government hospital	41	80.03
Private clinic	10	19.61
*Working experience*		
10–15 years	14	27.5
16–20 years	33	64.7
> 20 years	4	7.84
*Terminal education*		
FCPS (*part 1 or 2*)	39	76.5
MBBS	12	23.5
*Designation*		
Professor	12	23.53
Associate Professor	4	7.84
Assistant Professor	17	33.33
Registrar	6	11.76
Medical Officer	2	3.92
General Practitioner	10	19.61
*Satisfied with drug promotion activities*		
No	46	90.20
Yes	5	9.80

Besides, most of the MDs were males (88.24%), working in public hospitals (80.03%), more than 80% had working experience of 10–20 years and had completed FCPS (80.03%) either part 1 or both (Table 1). Among MDs, 33.3% were working as assistant professors, 23.53% as professors and 11.76% as registrars, while only 19.6% were GPs. Almost 90.2% doctors were not satisfied with current drug promotional strategies (Table 1).

### *Perception about ethical promotion of pharmaceuticals among medical representatives (*MRs*)*

As shown in Table 2, majority of MRs of both NCs and MNCs perceived that companies provided quality information about their products (NCs; 91.7%, MNCs; 96.8%) and always tag their samples “*not for sale*” (NCs; 88.3%, MNCs; 90.4%). The MRs of MNCs were more likely to believe that their companies follow ethical guidelines (OR; 6.37, *p* = 0.0005), aware of IFPMA (OR; 6.45, *p* = 0.0005), WHO (OR; 5.31, *p* = 0.0005), national codes of ethics (OR; 5.84, *p* = 0.0005) and Pakistan Medical Research Council (PMRC) codes of ethics (OR; 5.04, *p* = 0.0005). Regarding promotional practices, MRs believed that MNCs were more likely to provide accurate and scientifically valid data on products (OR; 4, *p* = 0.007), respect privacy of the data (OR; 2.34, *p* = 0.049), trained their MRs on ethical promotion (OR; 6.68, *p* = 0.0005) and had adequate controls and accountability measures for the samples provided to MDs (OR; 1.96, *p* = 0.047). Conversely, the MRs of NCs perceived that company obliged MDs by sending them to international conferences (OR; 0.15, *p* = 0.0005), sponsored social and entertainment activities of MDs in international conferences (OR; 0.123, *p* = 0.0005), always willing to bear the cost of accompanying individuals (OR; 0.09, *p* = 0.0005), offered cash or cash equivalents (OR; 0.103, *p* = 0.0005) and were fulfilling MD’s request for any facilitation or gifts (OR; 0.145, *p* = 0.0005) (Table 2).

**Table 2:** Perception of medical representative (MRs) on ethical promotion of pharmaceuticals

Questions	Overall (%)		Medical representatives (MRs) (%)		OR (CI)	*p* values
	No	Yes	NCs, *n* = 205	MNCs, *n* = 125		
Company provides information of high standards on product quality, safety, efficacy according to the standards framed by regulatory authorities	21 (6)	309 (94)	188 (91.7)	121 (96.8)	2.73 (0.9–8.3)	0.066
Company follows ethical guidelines for promotion and marketing of drug products	48 (15)	282 (85)	162 (79.0)	120 (96.0)	6.37 (2.5–16.5)	0.0005**
Aware of IFPMA guiding principles of ethical conduct and promotion	133 (40)	197 (60)	92 (44.9)	105 (84.0)	6.45 (3.7–11.2)	0.0005**
Aware of WHO ethical codes and promotion	119 (36)	211 (64)	105 (51.2)	106 (84.8)	5.31 (3.1–9.3)	0.0005**
Aware of Pakistan national code of ethics	132 (40)	198 (60)	94 (45.9)	104 (83.2)	5.84 (3.4–10.1)	0.0005**
Aware of PMRC code of ethics	165 (50)	165 (50)	73 (35.6)	92 (73.6)	5.04 (3.1–8.2)	0.0005**
Company provides accurate, balanced and scientifically valid data on products	28 (8)	302 (92)	181 (88.3)	121 (96.8)	4.01 (1.4–11.8)	0.007*
Company respect private and personal data of patients or medical doctor	32 (10)	298 (90)	180 (87.8)	118 (94.4)	2.34 (0.9–5.6)	0.049*
Company provides training to MRs on ethical promotion of their products	79 (24)	251 (76)	135 (65.9)	116 (92.8)	6.68 (3.2–13.9)	0.0005**
Company sponsors or organizes events for medical doctors outside Pakistan other than international conferences	179 (54)	151 (46)	127 (62.0)	24 (19.2)	0.15 (0.1–0.3)	0.0005**
Company sponsors social or entertainment activities of medical doctors in international conferences	178 (54)	152 (46)	130 (63.4)	22 (17.6)	0.123 (0.1–0.21)	0.0005**
Company is always willing to bear the costs of individuals accompanying the invited medical doctor on conferences	175 (53)	155 (47)	135 (65.9)	20 (16.0)	0.09 (0.1–0.2)	0.0005**
Company offers payments in cash or cash equivalents to medical doctors	233 (71)	97 (29)	88 (42.9)	9 (7.2)	0.103 (0.1–0.21)	0.0005**
Company always mark the samples with “Not for sale” tag	36 (11)	294 (89)	181 (88.3)	113 (90.4)	1.25 (0.6–2.6)	0.551
Company has the adequate system of controls and accountability for samples provided to medical doctors	51 (15)	279 (85)	167 (81.5)	112 (89.6)	1.96 (0.9–3.8)	0.047*
Company fulfils health care provider’s request for any facilitation or gift	151 (46)	179 (54)	146 (71.2)	33 (26.4)	0.145 (0.9–2.4)	0.0005**

*MNCs* multinational companies, *NCs* national companies

*p* values: *p* 0.05–0.002 = *, *p* ≤ 0.001 = **

### *Perception about ethical promotion of pharmaceuticals among medical doctors (*MDs*)*

As evident in Table 3, majority of MDs scored higher in favor of MNCs that denoted positive perception about their promotion, such as MNCs provide products of high quality, safety and efficacy (NC; 3.61 ± 1.16, MNCs; 4.35 ± 0.68, *p* = 0.0001), follow ethical guidelines in promoting their products (NC; 1.92 ± 0.75, MNCs; 4.43 ± 0.61, *p* = 0.0001), provide accurate balanced and scientifically valid data on products (NC; 1.98 ± 0.64, MNCs; 4.41 ± 0.61, *p* = 0.0001), provide information and scientific data with valid references (NC; 2.29 ± 0.67, MNCs; 4.41 ± 0.61, *p* = 0.0001). Conversely, MDs agreed/strongly agreed to perceptions implying un-ethical promotion of pharmaceuticals by NCs, such as organize events for MDs outside Pakistan other than international congress (NC; 4.1 ± 0.71, MNCs; 1.88 ± 0.86, *p* = 0.0001), finance social or entertainment activities for MDs in international congress (NC; 4.19 ± 0.63, MNCs; 1.65 ± 0.72, *p* = 0.0001), pay cost of individuals accompanying MDs on conferences (NC; 4.3 ± 0.58, MNCs; 1.67 ± 0.47, *p* = 0.0001), fulfil MD’s request for any facilitation or gifts (NC; 4.35 ± 0.62, MNCs; 1.96 ± 0.51, *p* = 0.0001), emphasize more on doctor-company sales contracts rather than doctor-patient suitability as per patient’s needs (NC; 4.27 ± 0.66, MNCs; 2.06 ± 0.9, *p* = 0.0001), more focused on selling tactics rather than product usage in the right indication (NC; 4.29 ± 0.67, MNCs; 1.67 ± 0.49, *p* = 0.0001) and always looking for MDs who prefer to write company products at the expense of certain benefits (NC; 4.35 ± 0.77, MNCs; 2.05 ± 0.33, *p* = 0.0001) (Table 3).

**Table 3:** Perception of medical doctors (MDs) on ethical promotion of pharmaceuticals

Questions on perception about ethical promotion	Medical doctors (MDs), *n* = 51 (5-point Likert score)		*p* values
	NCs (mean ± SD)	MNCs (Mean ± SD)	
Provides products of high quality, safety, efficacy according to the standards framed by regulatory authorities	3.61 ± 1.16	4.35 ± 0.68	0.0001*
Follows ethical guidelines for promoting and marketing of their products	1.92 ± 0.75	4.43 ± 0.61	0.0001**
Provides accurate, balanced and scientifically valid data on products	1.98 ± 0.64	4.41 ± 0.61	0.0001**
Provide information and scientific data with valid reference	2.29 ± 0.67	4.41 ± 0.61	0.0001**
Organize events for medical doctors outside Pakistan other than international congress	4.1 ± 0.71	1.88 ± 0.86	0.0001**
Finance social or entertainment activities for medical doctors in international congress	4.19 ± 0.63	1.65 ± 0.72	0.0001**
Always mark the samples with “Not for sale” tag	3.58 ± 1.25	1.61 ± 0.70	0.0001**
Pay cost of individuals accompanying medical doctors on conferences	4.3 ± 0.58	1.67 ± 0.47	0.0001**
Offers payments in cash or cash equivalents to medical doctors	4.35 ± 0.48	4.18 ± 0.79	0.567
Fulfils medical doctor’s request for any facilitation or gifts	4.35 ± 0.62	1.96 ± 0.51	0.0001**
More emphasis on doctor-company sales contracts rather than doctor-patient suitability as per the needs of the patients	4.27 ± 0.66	2.06 ± 0.9	0.0001**
More focused on selling tactics rather than product usage in right indication	4.29 ± 0.67	1.67 ± 0.49	0.0001**
Always looking for doctors who prefer to write company products at the expense of certain benefits	4.35 ± 0.77	2.05 ± 0.33	0.0001**

p-values: *p* 0.05–0.002 = *, *p* ≤ 0.001 = **

### Priorities of pharma companies in promoting pharmaceuticals; medical representative’s perspectives

Out of total, 42.4% and 53.6% MRs of NCs and MNCs, respectively, answered that patient’s well-being was the first priority of pharma companies. This was followed by maximum sales (NCs; 36.1%, MNCs; 30.4%), company’s repute (NCs; 10.2%, MNCs; 10.4%) and market position (NCs; 11.2%, MNCs; 5.6%). When asked about the purpose of the symposia, congress and scientific meetings for MDs, the MRs of both companies believed that these were aimed at providing the scientific information (NCs; 43.4%, MNCs; 72.8%), facilitate MDs (NCs; 32.2%, MNCs; 20%) and promote sales (NCs; 24.4%, MNCs; 7.2%) (Additional file 1: Table S1).

### Barriers to ethical promotion of pharmaceuticals; medical representative’s and doctor’s perspectives

The MR’s and MD’s perspectives regarding barriers to ethical promotion of pharmaceuticals in Pakistan are summarized in Table 4 and 5. Majority of MRs working for MNCs scored higher for majority of the questions pertaining to barriers, such as lack of external accountability (NCs; 3.66 ± 1.33, MNCs; 4.17 ± 1.1, *p* = 0.0003), price war (NCs; 3.73 ± 1.26, MNCs; 4.18 ± 0.97, *p* = 0.0007), to maximize the profit (NCs; 3.83 ± 1.19, MNCs; 4.22 ± 0.99, *p* = 0.003), lack of research (NCs; 3.62 ± 1.33, MNCs; 4.28 ± 0.96, *p* = 0.0001), low quality products need unethical push (NCs; 3.67 ± 1.35, MNCs; 4.38 ± 0.96, *p* = 0.0001), job insecurity in case of below target sales (NCs; 3.61 ± 1.34, MNCs; 4 ± 1.19, *p* = 0.008), materialistic approach by the MDs lacking ethical and moral values (NCs; 3.57 ± 1.2, MNCs; 3.88 ± 1.26, *p* = 0.033), managers not only condone but also encourage unethical promotion (NCs; 3.31 ± 1.34, MNCs; 3.75 ± 1.28, *p* = 0.003) and low salaries compel MRs to opt unethical promotion for incentives on sales (NCs; 3.57 ± 1.23, MNCs; 3.96 ± 1.22, *p* = 0.006) (Table 4).

**Table 4:** Barriers to ethical promotion of pharmaceuticals in Pakistan; MR’s perspective

Questions on barriers in ethical promotion	Medical representatives (MRs) (5-point Likert score)		*p* values
	NCs, *n* = 205 (Mean ± SD)	MNCs, *n* = 125 (Mean ± SD)	
Lack of external accountability can be a reason of unethical practices	3.66 ± 1.33	4.17 ± 1.1	0.0003*
Doctors are incentivized to generate business by undue investigation and overtreatment of patients who are at their mercy, medically and financially	3.56 ± 1.23	3.82 ± 1.26	0.077
Price war in Pharmaceuticals	3.73 ± 1.26	4.18 ± 0.97	0.0007**
To maximize the profit	3.83 ± 1.19	4.22 ± 0.99	0.003*
Lack of Research and Study	3.62 ± 1.33	4.28 ± 0.96	0.0001**
Company pressure to achieve sale targets	3.88 ± 1.24	4.02 ± 1.21	0.318
Low quality products need unethical push	3.67 ± 1.35	4.38 ± 0.96	0.0001**
Job insecurity if sales below target	3.61 ± 1.34	4 ± 1.19	0.008*
Non-existence of Doctor-Patient-Pharmacist Loop	3.55 ± 1.27	3.98 ± 1.11	0.003*
Prescribing by Brand names	3.8 ± 1.23	4. ± 1.11	0.097
Doctors are materialistic lacking ethical and moral values	3.57 ± 1.2	3.88 ± 1.26	0.033*
Sales managers not only condone unethical promotions of MRs but also encourage it	3.31 ± 1.34	3.75 ± 1.28	0.003**
Low salaries, thus for incentives, MRs opt un-ethical promotion	3.57 ± 1.23	3.96 ± 1.22	0.006*
Junior doctors use Pharma companies to promote their business (clinic renovation, foreign trips) and clinical practice (speakers at the seminar, free camps)	3.94 ± 1.14	3.98 ± 1.17	0.747

*MNCs* multinational companies, *NCs* national companies, *MR* medical representative (s)

*p* values: *p* 0.05–0.002 = *, *p* ≤ 0.001 = **

**Table 5:** Barriers to ethical promotion of pharmaceuticals in Pakistan; MD’s perceptive

Questions on barriers in ethical promotion	Medical doctors (MDs), *n* = 51 (%)					*p* values
	Strongly disagree	Disagree	Neutral	Agree	Strongly agree	
Lack of external accountability can be a reason of unethical practices	0 (0)	0 (0)	0 (0)	31 (61)	20 (39)	0.35
Doctors are incentivized to generate business by over investigation and treatment of patients at their mercy, medically and financially	7 (13.7)	8 (15.7)	10 (19.6)	15 (49)	11 (21.6)	0.42
Price war among pharmaceuticals	0 (0)	0 (0)	0 (0)	28 (55)	23 (45)	0.24
Out of competition companies resort to unethical promotion to maximize the profits	0 (0)	0 (0)	0 (0)	25 (49)	26 (51)	0.66
Lack of interests in research and study	0 (0)	0 (0)	0 (0)	25 (49)	26 (51)	0.21
Company’s pressure to achieve sale targets	0 (0)	0 (0)	0 (0)	21 (41)	30 (59)	0.59
Low quality products need unethical push	0 (0)	0 (0)	0 (0)	21 (41)	30 (59)	0.27
Job insecurity if sales targets not met	0 (0)	0 (0)	0 (0)	17 (33)	34 (67)	0.42
Non-existence of doctor-patient-pharmacist loop	0 (0)	0 (0)	0 (0)	20 (39)	31 (61)	0.35
Prescribing by brand names	0 (0)	4 (8)	1 (2)	24 (47)	22 (43)	0.13
Doctors are materialistic lacking ethical and moral values	1 (2)	13 (25)	1 (2)	16 (31)	20 (39)	0.56
Sales managers not only condone unethical promotion by MRs but also encourage it	0 (0)	0 (0)	0 (0)	23 (45)	28 (55)	0.02*
Low salaries, for incentives MRs opt un-ethical promotion	0 (0)	0 (0)	0 (0)	23 (45)	28 (55)	0.24
Junior doctors use pharma companies to promote their business (clinic renovation, foreign trips) and clinical practice (speakers at the seminar, free camps)	2 (4)	5 (10)	1 (2)	16 (31)	27 (53)	0.29

Majority of the MDs, public and private, either agree (A) or strongly agree (SA) that lack of external accountability (A; 61%, SA; 39%), price war among pharmaceuticals (A; 55%, SA; 45%), out of competition companies resort to unethical promotion to maximize the profits (A; 49%, SA; 51%), lack of interests in research and study (A; 49%, SA; 51%), company’s pressure to achieve sale targets (A; 41%, SA; 59%), low quality products need unethical push (A; 41%, SA; 59%), job insecurity if sales targets not met (A; 33%, SA; 67%), non-existence of doctor-patient-pharmacist loop (A; 39%, SA; 61%), sales managers not only condone unethical promotion by MRs but also encourage it (A; 45%, SA; 55%) and low salaries but incentives on achieving sales targets (A; 45%, SA; 55%) were the main barriers in ethical promotion (Table 5). The MDs posed barriers included, incentivized MDs that generate business by over investigation and treatment of patients at their mercy (medically and financially), materialistic mindset of MDs and junior MD’s inclination to promote their business (clinic renovation, foreign trips) and clinical practice (speakers of the seminar, free camps) (Table 5).

## Discussion

The pharmaceutical market of Pakistan is one of the emerging markets among the developing countries, worth about $3.2 billion [13]. The pharmaceutical companies promote their products to doctors, patients and facilities for health care and to reinforce sales revenues, but the purposeful desire to make profits and to maximize the market share invariably affect their promotional strategies. The present study is the first study from Pakistan that assessed the perception of and barriers to ethical promotion of pharmaceuticals in Pakistan by including the perspectives of both MRs and MDs from seven major cities of all four provinces of Pakistan. The study revealed that majority NCs and MNCs hired MRs with Bachelor of Science, who in routine paid 10 -15 visits/day to MDs, while majority of the MDs, qualified FCPS part 1 or both, worked in public hospitals and were not satisfied with drug promotional practices. Majority of the MRs perceived that MNCs follow certain guidelines on ethical promotion of pharmaceuticals compared to NCs. Likewise, MDs also perceived that most of the un-ethical practices to appease MDs for profits were patronized by NCs.

We observed that majority of the MRs of MNCs and NCs were trained to pay from less than 5 to more than 15 visits per day to the MDs. Literature evidences suggest that the frequency of MR’s visits to MD’s clinic is one of the major factor that influence the prescribing practices of MDs by impacting the decision making process, probably by affecting MD’s prescribing memory amendable to number of visits [14, 15]. Another study also revealed that persuasion by MRs might have far reaching impact on the prescribing behaviors of MDs [16]. Besides, the MRs were trained enough and encouraged to interact at personal level with the MDs, which they do so by paying regular visits—as majority of the MDs believed that they develop soft corner (liking) for the MRs who visit them regularly [17]. This aggressive promotion can jeopardize professional ethics and may influence or impel the prescriber to prescribe irrational medications affecting patient’s outcomes and incurred finances—echoing unethical practices [18]. We observed that compared to MRs of NCs, MRs of MNCs were more likely to endorse ethical promotion of pharmaceuticals, aware of IFPMA, WHO, PMRC and Pakistan’s national codes of ethics, believed that their companies provided scientific information of higher standard and quality, and provided training on ethical promotion of pharmaceuticals. Similarly in another study from Karachi, Pakistan, it was observed that as compared to NCs, MNCs are more likely to follow the promotional codes for advertisement [19]. Conversely, MRs of NCs were less likely to be aware of various codes of ethics of pharmaceutical promotion and only 65.9% acceded to the fact that companies provide training on ethical drug promotion. These differences could be due to set procedures in place for the approval of communications in MNCs—scientific in majority of the cases, against the applicable laws, regulations and codes by a qualified medical doctor or a pharmacist [20]. While, in NCs such approvals and communications, are disposed of by a science graduate rather than health care professional, doctor or a pharmacist, who, as a health care professional obliged to ensure patient welfare and abide by the codes of conduct of their professional bodies. Furthermore, MDs corroborated the perspectives of MR’s working for MNC and believed that MNCs followed ethical promotional practices, provided products of high-quality standards and accurate scientific information. In contravention, MDs believed that NCs tend to finance social and entertainment activities, gave all kind of facilitations and gifts to MDs, emphasized more on doctor-company sale contracts, more focused on products sales rather than its usage in the right indication and always looking for MDs who prescribe company products in exchange of certain benefits. Nevertheless, blaming solely to the pharma companies could echo an inequitable justice. A study from Karachi, Pakistan revealed that almost 36% medical doctors admitted that they demanded gifts from MRs, while 63.8% MRs were of the view that prescribers demand unethical inducements like gifts, product samples, foreign trips, clinical renovation and expensive gifts in form of cars [9]. In this context, several reports from Pakistan provided ample evidences that both MDs and pharma companies are involved in unethical promotional practices prevalent in Pakistan, such as a study from Nishtar Hospital Multan highlighted the misuse of samples by the doctors [21], a study from Sukker Division of Pakistan revealed that both pharmaceutical companies and doctors are equally responsible for unethical promotional activities [22], another study from Rawalpindi, suggested the involvement of both MRs & MDs in unethical promotion of drugs in Pakistan [23]. Nonetheless, the quality interaction between the prescriber and the MRs may be necessary to equip health care professional with leading-edge drug related information. However, there is also evidence that these interactions are associated with poorer prescribing practices [24, 25].

Regarding barriers in ethical promotion of pharmaceuticals, compared to NCs, MRs of MNCs scored higher and acceded to several barriers faced by pharma companies, such as lack of external accountability, price wars, pressure to achieve sale targets, excessive push to sell low quality products, materialistic mindset of doctors, sales managers condone unethical promotion and low salaries driven unethical promotion to achieve incentivized targets. While, MRs of NCs and MNCs believed that junior doctors exploit pharma companies to promote their businesses. Majority of MDs also believed that the barriers mentioned above were the foremost barriers in ethical promotion of pharmaceuticals—including materialistic mindset of MDs. A previous study from Pakistan suggested that majority of the physicians did not consider the current pharma marketing practices as unethical rather considered educational seminars and associated activities as beneficial for doctors, yet accepting that the current drug promotion practices are not following any ethical codes or standards [26].These findings and the results mentioned above clearly suggested that both MRs and MDs are cognizant about the causes of unethical promotion of pharmaceuticals in Pakistan, nonetheless, both stakeholders, pharma companies, out of profits, and MDs, out of free services, became habitual to the unethical and unnecessary symbiotic relationship without any attention to their professional duties towards patient welfare and well-being [18, 26, 27].

### Policy implications and recommendations

The ministry of national health services regulation and coordination, government of Pakistan, in consultation with drug regulatory authority of Pakistan (DRAP) under statutory notification on June 2017, provided code of conduct for ethical marketing to health care professionals. To our knowledge, as of today, no real time implementation is perceptible at public or private health care facilities. As per the codes of ethics, pharma companies can provide modest meals and educational items to business discussions and MDs, respectively. Moreover, companies may engage health care professionals to provide services that support research and development to advance in medical science, develop new technologies, improve existing products and services, educate on the safe and effective use of company products or enhance the quality and efficacy of patient care. Companies may provide training and education of Healthcare Professional on the safe and effective use of Company products, including “hands-on” training sessions, cadaver workshops, wet lab sessions, live surgeries, lectures and presentations. However, pharma companies should not provide any gifts or sponsor entertainment activities of MDs. But according to this notification, not a government executive, rather a senior executive appointed by the company will oversee all these practices and warrant their compliance to these codes of ethics—purely against the spirit of ethical promotion. With regards to contravention and punishment, whosoever himself or by any other person on his behalf contravenes with the provisions of the DRAP Act 2012 and regulations made there under shall be punishable as provided for in Schedule II and III of the DRAP Act 2012. Under Schedule II, no person shall himself or by any other person on his behalf advertise, distribute therapeutic good as sample and print label for the therapeutic goods, failure to comply would result in imprisonment for a term up to five years and with fine up to five hundred thousand rupees (~ $3000). Nonetheless, to our knowledge, not a single individual has been penalized for misleading advertisement, yet there have been several reports where the culprit has been penalized for distributing doctor’s sample and for printing fake labels—suggesting poor implementation of this act with regards to unethical promotion of pharmaceuticals in Pakistan. While under Schedule III, whoever himself or by any other person on his behalf imports, export, manufacture or sale any spurious, counterfeit therapeutic good without a license shall be punishable with imprisonment for a term which may extend to seven years, or with fine which may extend to five hundred thousand rupees (~ $3000) or with both.

In other countries of South Asian region, like India and Bangladesh, the situation is not much different. In India, the principal legislation that regulates the pharmaceutical industry, i-e., Drug and Cosmetic Act, 1940, does not cover much about the drug promotion regulations, i-e., do’s and don’ts of promotion to health care professionals (HCPs), thus, back in 2011 and later revised in 2014, Uniform Code of Pharmaceutical Marketing Practices (UCPMP) and Organization of Pharmaceutical Procedures of India (OPPI) was introduced with the intent to guide and to set standards of interactions between the pharma industry and HCPs. However, despite the regulations in place for the medical doctors by Medical Council of India and pharma industry by UCPMP, the former seems to be ambiguous, non-comprehensive and poorly implemented, while the latter seems to be self-regulatory codes lacking regulatory and legal binding [28, 29]. Likewise, in 1994, Bangladesh formulated the Code of Pharmaceutical Marketing Practices (CPMP) to promote ethical marketing of pharmaceutical products but failed to curtail the misleading claims made in drug advertisements [17, 30].

In Pakistan, it’s a matter of grave concern that majority of MRs working for NCs were oblivious of WHO, IFPMA and national codes of ethics regarding pharmaceutical promotion. This suggested that the pharmaceutical drug promotion practices are unattended and unaccountable for any misconduct affecting patient’s life and out of pocket finances. Thus, utmost attention should be paid to implement and maintain ethical standards of drug promotion by the health system regulators, health practitioners and professional organizations. The policy makers and regulators should ensure stricter legislation with regards to unethical promotion with mechanisms of policy implementation, regular monitoring, screening of printed promotional material and punitive fines for offenders. There should be written protocols on ethical marketing for awareness with training programs in place for all health workers. There should be courses, during undergraduate training, on ethical promotion of drugs dealing with the art of critical appraisal of drug promotion literature to confirm product claims. Importantly, Pakistan should adopt standard codes of ethics on pharmaceutical marketing, such as WHO or IFPMA with inhouse modifications in line with the needs of local health care and pharmaceutical market. However, critics of the way that pharmaceutical promotion is regulated believe that the WHO code is significantly stronger than the IFPMA one [31, 32]. Besides, professional bodies and councils should audit the conduct of their members and must have system of periodic assessment of drug related knowledge of practicing MDs.

### Study limitations

The study has several limitations, the cross-sectional design of the study did not allow the documentation over an extended period of time. The response rate of MRs was not optimal because we were unable to visit them personally due to limited resources. Data obtained through self-administered questionnaires are self-reported and might be subject to bias. Besides, the gathered information is purely based on MDs and MRs self-sensed responses rather than the actual observation by an observer.

## Conclusion

Taken together, these data suggest that compared to MRs of NCs, majority of the MRs working in MNCs were more likely to follow and own adequate information about different codes of ethics in ethical promotion of pharmaceuticals. While, NCs tend to facilitate MDs in form of gifts, social and entertainment activities, local and abroad. Similar perception was upheld by the MDs regarding ethical promotion except one common tag that both MNCs and NCs offer cash payments or equivalents to MDs. Moreover, MRs of NCs and MNCs agreed or strongly agreed that pressure to achieve sales targets, incentivized MDs, sales linked job security, MD’s materialistic mindset, brand prescribing and junior doctor’s reliance on companies for their businesses were the predominant barriers. Likewise, majority of the MDs agree or strongly agree to the above-mentioned barriers in addition to lack of external accountability and blatant condonation of high-ups for such activities.

## Supplementary information

**Additional file 1: Table S1.** Pharma companies’ priorities in promoting pharmaceuticals, medical representative’s perspectives.

## Data Availability

The datasets used and analyzed in this study can be available from the corresponding author on reasonable request.

## Abbreviations

PCs: Pharmaceutical companies
MNCs: Multi-national companies
NCs: National companies
MRs: Medical representatives
MDs: Medical doctors
DRAP: Drug regulatory authority of Pakistan
IFPMA: International Federation of Pharmaceutical Manufacturers and Associations
PMRC: Pakistan medical research council
OTC: Over the counter
GPs: General practitioners
FCPS: Fellow of college of physicians and surgeons
A: Agree
SA: Strongly agree
