# Supplementary material for: Perceptions of and barriers to ethical promotion of pharmaceuticals in Pakistan: perspectives of medical representatives and doctors
Source: BMC Med Ethics. 2021 Jan 4;22:2. doi: 10.1186/s12910-020-00569-0 (PMC7783997; doi:10.1186/s12910-020-00569-0)
Supplement: Supplementary file 1 — Additional file 1: Table S1. Pharma companies’ priorities in promoting pharmaceuticals, medical representative’s perspectives. [file 12910_2020_569_MOESM1_ESM.docx]

**Additional file 1: Table S1.** Pharma companies’ priorities in promoting pharmaceuticals, medical representative’s perspectives

| **Questions** | | **Priority** | **Pharmaceutical companies** | | ***p*-value** |
| --- | --- | --- | --- | --- | --- |
|  |  |  | **NCs, *n*=205 (***%***)** | **MNCs, *n*=125 (***%***)** |  |
| First priority of your Pharmaceutical company | | ***Well-being of patient*** | 87 (42.4) | 67 (53.6) | 0.138 |
|  |  | ***Maximum Sale*** | 74 (36.1) | 38 (30.4) |  |
|  |  | ***Company's repute*** | 21 (10.2) | 13 (10.4) |  |
|  |  | ***Market Position*** | 23 (11.2) | 7 (5.6) |  |
| The purpose and focus of all symposia, congresses and other promotional, scientific or professional meetings organized or sponsored by your company for medical doctors is to | | ***Provide scientific information*** | 89 (43.4) | 91 (72.8) | 0.001** |
|  |  | ***Facilitate doctor*** | 66 (32.2) | 25 (20) |  |
|  |  | ***Promote sales*** | 50 (24.4) | 9 (7.2) |  |
|  | ***p-values:*** *p* 0.05 – 0.002 = *, *p* ≤ 0.001 = ** | | | | |
